# Supplementary material for: Comparative multiomics analysis of cell physiological state after culture in a basket bioreactor
Source: Sci Rep. 2022 Nov 23;12:20161. doi: 10.1038/s41598-022-24687-4 (PMC9686226; doi:10.1038/s41598-022-24687-4)
Supplement: Supplementary file 1 — Supplementary Information 1. [file 41598_2022_24687_MOESM1_ESM.zip › raw data/Transcriptomic raw data/7.AS/2.dasplot/TE1vsTC1/MXE/19_LOC103231469_NW_023666059.1_22738241_22738680_-@NW_023666059.1_22737505_22737621_-@NW_023666059.1_22727059_22727154_-@NW_023666059.1_22724584_22724698_-.pdf]

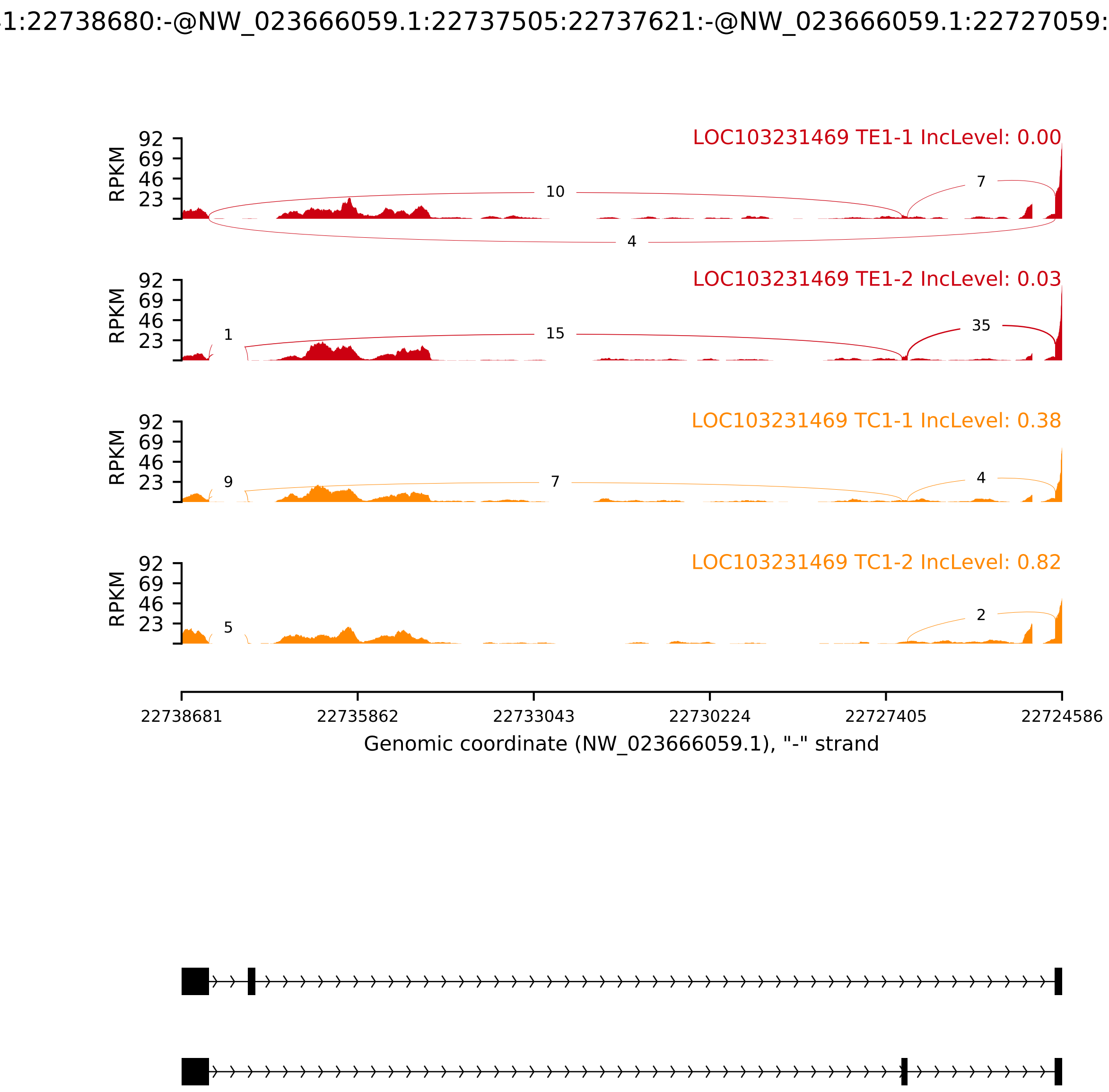

1:22738680:-@NW_023666059.1:22737505:22737621:-@NW_023666059.1:22727059:22727154:-@NW_0236660
LOC103231469 TE1-1 IncLevel: 0.00
LOC103231469 TE1-2 IncLevel: 0.03
LOC103231469 TC1-1 IncLevel: 0.38
LOC103231469 TC1-2 IncLevel: 0.82
RPKM
92
69
46
23
10
7
4
1
15
35
9
7
4
5
2
22738681
22735862
22733043
22730224
22727405
22724586
Genomic coordinate (NW_023666059.1), "-" strand
